# Adaptive bandwidth kernel density estimation for next-generation sequencing data

**DOI:** 10.1186/1753-6561-7-S7-S7

**Published:** 2013-12-20

**Authors:** Parameswaran Ramachandran, Theodore J Perkins

**Affiliations:** 1 Ottawa Hospital Research Institute, Regenerative Medicine Program, K1H 8L6, Ottawa, Canada; 2 University of Ottawa, Department of Biochemistry, Microbiology and Immunology, Faculty of Medicine, K1H 8M5, Ottawa, Canada

## Abstract

**Background:**

High-throughput sequencing experiments can be viewed as measuring some sort of a "genomic signal" that may represent a biological event such as the binding of a transcription factor to the genome, locations of chromatin modifications, or even a background or control condition. Numerous algorithms have been developed to extract different kinds of information from such data. However, there has been very little focus on the reconstruction of the genomic signal itself. Such reconstructions may be useful for a variety of purposes ranging from simple visualization of the signals to sophisticated comparison of different datasets.

**Methods:**

Here, we propose that adaptive-bandwidth kernel density estimators are well-suited for genomic signal reconstructions. This class of estimators is a natural extension of the fixed-bandwidth estimators that have been employed in several existing ChIP-Seq analysis programs.

**Results:**

Using a set of ChIP-Seq datasets from the ENCODE project, we show that adaptive-bandwidth estimators have greater accuracy at signal reconstruction compared to fixed-bandwidth estimators, and that they have significant advantages in terms of visualization as well. For both fixed and adaptive-bandwidth schemes, we demonstrate that smoothing parameters can be set automatically using a held-out set of tuning data. We also carry out a computational complexity analysis of the different schemes and confirm through experimentation that the necessary computations can be readily carried out on a modern workstation without any significant issues.

## Introduction

High-throughput sequencing (HTS) has become a central technology in genome-wide studies of protein-DNA interactions, chromatin-state modifications, gene regulation and expression, copy number variations, etc. [1,2]. In many cases, such experiments can be viewed abstractly as attempting to measure a "signal" *f *that varies across the genome. For instance, if the DNA that is sequenced comes from chromatin immunopreciptation (ChIP) of a transcription factor, then the signal *f *is expected to have the highest amplitude in regions of the genome where the factor binds most strongly. If the sequenced DNA comes from reverse transcription of RNA, then *f *is expected to have the highest amplitude in regions of the genome that are most actively transcribed. Of course, experience with HTS technologies has shown that such genome-wide signals also reflect other biases or influences--due to, for example, sequencing, chromatin accessibility, mappability, etc. [3]. Techniques for correcting such biases are beginning to emerge [4,5]. Regardless, highthroughput sequencing continues to generate numerous important insights into the molecular networks that govern the cell.

Various analysis algorithms specialize in extracting biologically-relevant information from different types of HTS data. For example, peak-calling algorithms take mapped reads and attempt to identify regions of high enrichment (for review and some comparisons, see [3,6,7]). Some algorithms attempt to solve this problem generally, whereas others specialize in identifying punctate transcription-factor binding sites [8,9] or, conversely, broader regional enrichment, as is often seen in histone modification patterns [7,10]. Similarly, a raft of algorithms specializes in estimating gene expression, including the expression of alternative spliceoforms (e.g., [11-14]). While such approaches are clearly valuable, few deal directly with the problem of estimating the genomewide signal *f*.

Yet, there are many reasons to be interested in such a direct reconstruction. Perhaps the most straightforward is that reconstructing *f *is useful for visualization in genome browser tracks. Visualization of the signal allows biologists to sanity-check their data, compare different signals at an intuitive level, identify regions of interest, generate hypotheses, and so on [15]. Reconstruction also allows us to manipulate and combine different signals, for example by "subtracting" a background noise/control signal from a treatment signal. Indeed, there is some evidence from the peak-calling literature that true binding and background processes can be separated, leading to enhanced signal fidelity [10,16]. We contend that such issues have not been explored in the literature nearly as thoroughly as they should have been, in part because of a lack of focus on the more elementary problem of reconstructing genome-wide signals themselves.

**Figure 1:**
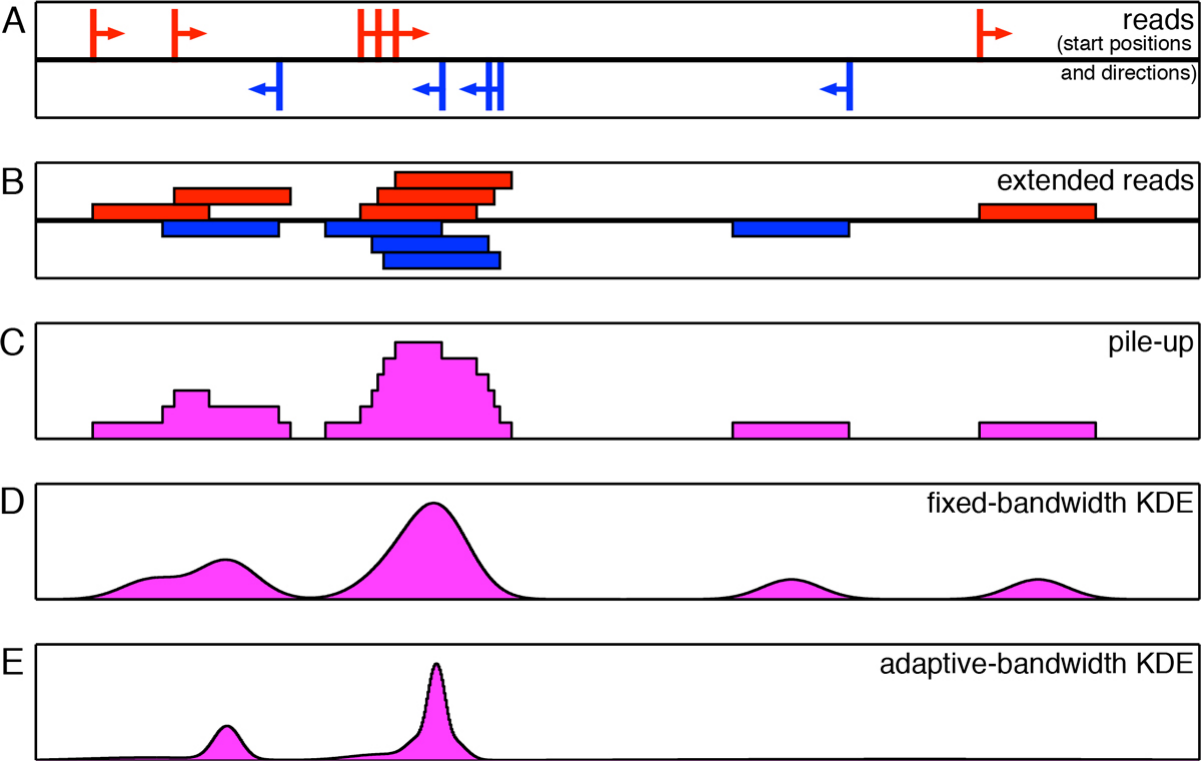
**Different approaches to genomic signal reconstruction**. (A) Reads in a portion of the genome. Positive-strand read starts are shown as spikes above the center line, while negative-strand read starts are below the center line. (B) Extending reads by the mean fragment length in the direction indicated by the strand to which they are mapped. (C) Pile-up curve obtained by adding together the extended read intervals, regardless of direction. (D) Fixed-bandwidth kernel density estimate (KDE) obtained using Gaussian kernels and after shifting each read position by half the mean fragment length in the read direction (positive or negative strand). (E) Adaptive-bandwidth KDE, also obtained using Gaussian kernels. This method creates greater smoothing in areas of low data density and emphasizes enriched regions more strongly.

The question then becomes, how can we best reconstruct the genome-wide signals measured by HTS experiments? One simple approach is a read "pileup" map. The details of computing a pile-up depend on whether DNA fragments are sequenced entirely or only partially and, in the latter case, also on whether they are sequenced partially from just one end (resulting in single-end reads) or from both ends (resulting in paired-end reads). In the case of a single-end dataset, which is probably the most common type of HTS dataset at present, the sequenced reads are mapped back to the genome to obtain their locations (Figure 1A). Then the positive-and negative-strand reads are either extended to the mean fragment length (Figure 1B) or shifted towards each other by half the mean fragment length. In the former case, the signal profile is built as an aggregation of the intervals representing the fragments (Figure 1C) [16,17]. In the latter case, the simplest way of building a profile is by using a moving histogram. This involves sliding a window of fixed width across the genome and counting the number of reads falling within the window as the window moves forward. Although such histograms have been implemented in various versions [8,18-20], in general, histograms are problematic as estimators because they are not smooth and the resulting estimates are strongly affected by the choice of histogram bin width.

An alternative and more accurate estimator is the *kernel density estimator *(KDE), where a kernel (e.g., Gaussian) of a chosen bandwidth (standard deviation) is centered at each sample point (a read), and the kernels are then summed to obtain the density estimate (Figure 1D) [21,22]. Intuitively, high-density regions would correspond to tall peaks due to the piling up of closely-spaced kernels. These KDE-based density estimates can be thought of as denoting the probability of finding a read at a given base pair location. QuEST [23], F-Seq [15], and Qeseq [7] apply this method to identify enriched regions in HTS data. Although the density estimates obtained by these algorithms are in general smoother and more accurate than those obtained using histograms, the bandwidths of the kernels are fixed and are chosen arbitrarily (QuEST uses 30 bp, Qeseq uses 150 bp, and F-Seq uses an indirect feature-length parameter to set bandwidth to typically a few thousand bp). The fact that the quality of the density estimates are very much dependent on the choice of the kernel bandwidth necessitates a more careful and methodical approach to bandwidth selection. In theory, a single optimal bandwidth can be systematically chosen for a given dataset using one of the popular plug-in or crossvalidation approaches [24-28]. However, the large genome sizes and the sparsity of HTS data make it a cumbersome process to estimate bandwidth in this manner. Even if achieved, a single bandwidth for the entire genome would not usually be sufficient for identifying enriched regions with a high degree of accuracy, owing to the widely varying spatial smoothness of the read distributions. Ideally, small bandwidths work best for high-density regions and large bandwidths work best for low-density regions. If the bandwidth is fixed for the entire genome, then it has to take a compromise value between the two extremes, thus limiting the accuracy of the resulting density estimate. In addition, the estimate would tend to have a large number of spurious local maxima corresponding to individual reads in low-density regions (Figure 1D). Due to these reasons, a fixed-bandwidth KDE is not the best choice for modeling the widely-varying distributions associated with ChIP-Seq or other types of HTS data.

An effective alternative is to use an adaptive scheme that utilizes local data features to dynamically adjust the density estimate to reflect variations in the underlying true density. *Adaptive-bandwidth *KDE, as the name suggests, achieves this by adapting (or varying) the kernel bandwidth according to the local characteristics of the data. Two types of adaptive-bandwidth KDEs have been investigated in the literature. First is the *balloon *estimator [29] where, for each estimation point, a bandwidth is first chosen and the estimate at that point is then computed as an average of the identically-scaled kernels evaluated at that point. The kernels are, of course, centered at the data points. Since the bandwidth is fixed for a given estimation point, this estimator, taken pointwise, behaves like a fixed-bandwidth KDE. Although the estimator has been shown to be promising in higher dimensions, it has serious drawbacks in the univariate and bivariate settings [30,31]. Most importantly, the estimate fails to integrate to one and, in certain situations, has a performance that is worse than that of the fixed-bandwidth KDE.

The second type of adaptive-bandwidth estimator is the *sample-point *estimator, where a bandwidth is selected for each data point instead of the estimation point [32]. The estimate $\hat{f}$ is then an average of differently-scaled kernels centered at the data points. When the kernel function is a density, $\hat{f}$ itself is a density. This type of estimator has been generally found to be a better performer than the balloon estimator [31,33,34], and is easily adaptable for HTS data. In addition, considering the large genomic sizes that are encountered, the estimate is simple and straightforward to compute since there are only as many kernels as the number of reads in the data. The only caveat, pointed out in [31], is a phenomenon referred to as "non-locality" where the estimate at a certain point can be affected by kernels corresponding to data very far away from it. However, in practice, this would not be an issue because, for the sake of computational feasibility, the kernel tails would have to be truncated after a reasonable number of standard deviations. This truncation would typically have no serious consequence as the values involved would be very small.

In this paper, we present an *adaptive-bandwidth *KDE for modeling the tag distributions of HTS data. The estimator automatically adjusts to the smoothness variations by choosing an appropriate local bandwidth for every read location, thereby leading to a much better estimate of the underlying distribution compared to that obtained using a fixed-bandwidth KDE. To the best of our knowledge, adaptive-bandwidth KDEs have not been considered for HTS data before. The method is inspired from the *sample-point *estimator [32], but has a number of new features that have been specifically developed to make it suitable for use in HTS data analysis. We consider three possibilities for the choice of the kernel function, namely, the square, triangular, and Gaussian distributions, and compare and evaluate their performance using a number of public datasets. For more detailed discussions on adaptive KDEs in general, the reader is referred to [29-31,33-35] and references therein.

## Methods

### Datasets

We compare different density estimation approaches on a suite of ten ENCODE single-end ChIP-Seq datasets available through the Gene Expression Omnibus. We downloaded the data in the form of BAM files, in which reads have already been mapped to positions in the human genome. We chose five datasets describing pulldowns for histone 3 with the following modifications: H3K27ac (GSM733718), H3K27me3 (GSM733748), H3K36me3 (GSM733725), H3K4me1 (GSM733782), and H3K4me2 (GSM733670). The other five datasets describe binding of the following transcription factors: BRCA1 (GSM935377), CTCF (GSM733672), GTF2F1 (GSM935581), RAD21 (GSM803466), and REST (GSM803365). For the sake of computational convenience, we restricted our attention to estimating the genomic signal on chromosome 1. In pilot studies we conducted, there were no significant differences in conclusions based on density estimation over the whole genome versus density estimation on just chromosome 1. By focusing on chromosome 1, our computations proceeded much faster. Hence, we first isolated the reads from chromosome 1, removed any duplicate reads, and then shifted positive and negative strand reads towards each other by one half the fragment length, which was estimated using the MaSC approach [5]. We took the starting positions of the resulting reads as data "points" for the purpose of density estimation, and sorted them in ascending order. Each dataset was thus reduced to a sorted list of positions *X *= (*x*_1_, *x*_2_, *. . *., *x_n_*).

### Fixed and adaptive bandwidth kernel density estimators

From a density-estimation perspective, the data *X *is viewed as being sampled from some unknown distribution *f*(*x*) on the genome. The idea is to estimate *f *using the sample data. We do this with a *kernel density estimator*, which is of the form

(1) $$\hat{f}\left(x\right)=\frac{1}{n}\overset{n}{\underset{i=1}{\sum }}K\left(x-x_{i},h_{i}\right)$$

Here, *x *is a *query point *at which we want to evaluate our estimate of *f*, *K *is a kernel function, *x_i _*is a sample data point, and *h_i _*is the *bandwidth *associated with *x_i_*. The kernel function *K*, for example, might be Gaussian in shape, with mean zero and standard deviation *h_i_*. The translated kernel, *K*(*x − x_i_*, *h_i_*), would then have mean *x_i_*.

In a *fixed-bandwidth *kernel density estimate, *h_i _*is equal to a constant value *h*, which may be chosen *a priori *or dependent somehow on the data. Intuitively, the larger *h *is, the more aggressively the data is smoothed, because the kernel function becomes broader for larger *h*. Below, we also experiment with blending fixed-bandwidth estimators with a uniform density. This creates a density of the form ${\hat{f}}_{\varepsilon }\left(x\right)=\left(1-\varepsilon \right)\hat{f}\left(x\right)+\varepsilon u\left(x\right)$, where *ε *∈ [0, 1], $\hat{f}\left(x\right)$ is the kernel density estimate of Eqn. 1, and *u*(*x*) is a uniform density over the genome. [In fact, because we are concerned with probabilities over integer base pair positions, we should be discussing probability *distributions *rather than probability *densities*. However, in keeping with the traditional terminology of these estimators, we will employ the term 'density' throughout.]

In an *adaptive-bandwidth *kernel density estimator, each *h_i _*is allowed to be different. We employ a variant of the *k*-nearest neighbor rule to assign the bandwidth *h_i_*. In the statistics literature, there are various schemes for assigning bandwidths. Perhaps the simplest and the most practical rule is to assign to point *x_i _*a bandwidth *h_i _*equal to the absolute distance from *x_i _*to its *k_th _*nearest neighbor [31]. We will call this the KNN1 rule. Intuitively, in regions of sparse data, the *k_th _*nearest neighbor will be far away, and so large bandwidths will be assigned, leading to aggressive smoothing of the signal. In dense regions, on the other hand, the *k_th _*nearest neighbor will be much closer, leading to small bandwidths, and thereby an accurate reconstruction of the signal. The choice of *k *allows us to indirectly control (to a certain degree) the bandwidth assigned to each point--large *k *values generally lead to large bandwidths, although the exact bandwidth assigned to each point depends on its proximity to its neighbors.

It turns out that the KNN1 rule has a minor problem which can be awkward in practice. Consider the situation where there are two regions of dense data with a sparse region in between. It may so happen that the bandwidths of all points (at least for some choices of *k*) may be set by points within the same dense region. Consequently, no points, including those at the inside edges of the dense regions, would be assigned a large bandwidth, wide enough to cover the span of the sparse region. Therefore, the points in the sparse region may each end up with a probability of zero. If we then evaluate a new set of data points on the density estimate, and a single point from this set happens to fall in the aforementioned sparse region, then the zero probability assigned to this point would result in the joint probability of the new set of points to be zero--all because of that single point in the sparse region. This "zero problem" is quite common with ChIP-seq datasets, where there are large numbers of very sparse regions.

To circumvent this problem, we propose a variant of KNN1 for assigning bandwidths, which we call KNN2. According to this rule, a point *x_i _*is assigned the same bandwidth as in the KNN1 rule unless all *k *of its nearest neighbors are on the same side (left or right). In that case, we instead take the bandwidth to be the distance to the single nearest point in the opposite direction. This rule ensures that the density estimate is nonzero everywhere (except possibly at the extreme ends of the range), thereby avoiding the zero problem.

### Kernel functions

We explore three possible shapes of the kernel function: Gaussian, square (also known as the Parzen window), and triangle (also known as the hat function). As a matter of computational convenience, we truncate the Gaussian distribution at ± 5 standard deviations. We interpret the bandwidth parameter *h *for each shape of the kernel function in such a way that, when viewed as a distribution in its own right, the standard deviation of that distribution is approximately equal to the bandwidth parameter. This ensures the greatest comparability of results from different kernel functions in experiments where we vary bandwidths or employ adaptive bandwidths. We also take care that each kernel function sums to one. As such, the three kernel functions we consider are

$$K_{g}\left(x,h\right)=\left\{\begin{array}{cc} c_{g}\left(h\right)\text{exp}\left(-x^{2}/2h^{2}\right) & \text{if}\;\text{|}x|\;\leq 5h\\ 0 & \text{if}\;\text{|}x|\;>5h \end{array}\right)$$

$$K_{sq}\left(x,h\right)=\left\{\begin{array}{cc} c_{sq}\left(h\right) & \text{if}\;\text{|}x|\;\leq \sqrt{3}h\\ 0 & \text{if}\;\text{|}x|\;>\sqrt{3}h \end{array}\right)$$

$$K_{tr}\left(x,h\right)=\left\{\begin{array}{cc} c_{tr}\left(h\right)\left(1-|x|/\sqrt{6}h\right) & \text{if}\;\text{|}x|\;\leq \sqrt{6}h\\ 0 & \text{if}\;\text{|}x|\;>\sqrt{6}h \end{array}\right)$$

Here, *c_g _*(*h*), *c_sq _*(*h*), and *c_tr _*(*h*) are normalizations that ensure, as a function of bandwidth, that each kernel sums to one.

## Results and discussion

### Effects of different density estimation schemes in genomic signal reconstruction

To help visualize the effects of different density estimation approaches on real data, we computed fixed- and adaptive-bandwidth Gaussian density estimates based on the Rad21 data in a window of chromosome 1. For fixed-bandwidth estimation, we considered two choices: *h *= 16 (close to the *h *= 15 default used in QuEST), and *h *= 362, which we show in the next subsection to be the optimal value according to the probability of held-out tuning data (at least when optimized over integer powers of $\sqrt{\;2}$). For adaptive bandwidth, we also considered two choices: *k *= 7, which is optimal according to a held-out tuning dataset, and *k *= 14, chosen to increase smoothing.

**Figure 2:**
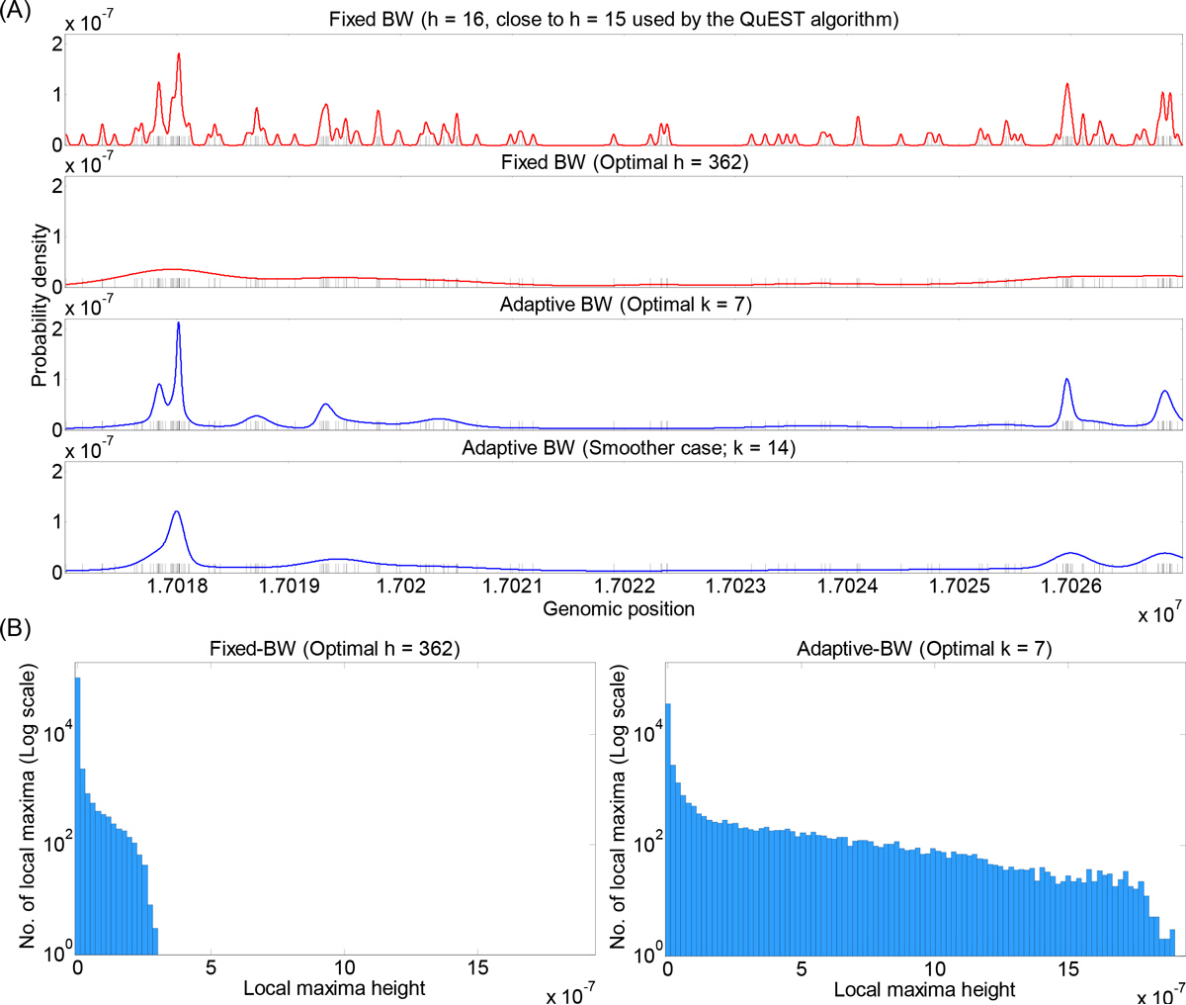
**Effects of different signal-reconstruction approaches**. (A) From top to bottom: fixed-bandwidth Gaussian estimators for a portion of the Rad21 data using *h *= 16 (close to the *h *= 15 that is default in the QuEST software) and *h *= 362 (which appears optimal based on evaluation of held-out tuning data), adaptive-bandwidth Gaussian estimators using *k *= 7 (optimal based on held-out tuning data) and *k *= 14 (double the optimal choice, and intended to obtain more aggressive smoothing). In each plot, the curve shows the reconstructed density. The short black vertical marks indicate the read positions. (B) Histograms of heights of local maxima for fixed-bandwidth Gaussian (*h *= 362) and adaptive-bandwidth Gaussian (*k *= 7). Note that the vertical axes are in log scale.

The results are shown in Figure 2A. The fixed bandwidth estimate with *h *= 16 includes many fluctuations across the window. Indeed, in data-sparse regions of the window, each data point (shown by a black mark) produces its own small bump in the curve, just as in our idealized example of Figure 1. Between these bumps, the density estimate is zero--although it is probably reasonable to expect that, if the experiments were repeated, reads may appear at other locations within the region of generally low signal. Still, the density estimate is highest where the most data can be found, and these strong peaks in the curve are readily picked out by the eye. With the larger bandwidth of *h *= 362, most fluctuations in the curve are smoothed away, leaving only broad swells where the data is most dense. This, correctly, eliminates the visual distraction of small fluctuations, although it also de-emphasizes the more dense regions and possible structure within them (such as possible multiple peaks). The adaptive bandwidth estimates eliminate small fluctuations for both choices of *k*, while still strongly emphasizing data-dense regions. The difference between the estimates corresponding to *k *= 7 and *k *= 14 has more to do with the fine structure of dense regions--questions such as "is an enriched region a single peak, or two or three separate peaks?" In the next section, we demonstrate that *k *(or *h*) can be optimized using held-out data in a tuning set. However, such questions of fine structure may also be studied by considering additional information, such as the locations of binding motifs for the factor, or signals in other ChIP-Seq datasets.

To quantitatively demonstrate the qualitative effects described above, we identified all strict local maxima for chromosome 1 in the fixed bandwith *h *= 362 and the adaptive bandwidth *k *= 7 curves. The curve *f *has a *strict *local maximum at position *b *if *f *(*b*) *> f *(*b − *1) and *f *(*b*) *> f *(*b *+ 1). While some local maxima correspond to regions of high read densities that are biologically significant, such as a transcription-factor binding site, other local maxima correspond to peaks of stand-alone kernel functions corresponding to individual reads that are likely to have no significance. Figure 2B shows histograms of the heights of these maxima. From the histograms, we see that the adaptive bandwidth estimator produces a much wider range of peak heights resulting from the fact that it strongly emphasizes data-dense regions. It also produces a smaller number of local maxima (NLM)--a trend that holds for most, though not all, of the datasets we have considered here (see Table 1). Unsurprisingly, we note a generally inverse relationship between the bandwidth *h *or the number of nearest neighbors *k *and the number of local maxima in the resulting density estimate. Nevertheless, all density estimates have tens of thousands of local maxima, considering that these results correspond only to chromosome 1. Therefore, when computed for the whole genome, the numbers can be expected to be much greater than the expected number of *bona fide *binding sites for a transcription factor.

**Table 1:** Optimized Parameters

Dataset	Fixed BW KDE			Adaptive BW KDE						
	
	Opt. *h*	NLM	CPU time (s)	Opt. *k*	NLM	CPU time (s) (Gau/Tri/Sq)				
H3K27ac	1024	40892	395	18	32357	1563	/	653	/	161
H3K27me3	2048	21476	230	16	12291	1513	/	620	/	128
H3K36me3	2048	21052	679	25	19035	2117	/	946	/	217
H3K4me1	1448	28264	240	7	43188	763	/	300	/	74
H3K4me2	724	55799	336	11	41147	934	/	376	/	83
BRCA1	1448	29078	861	24	33822	2158	/	842	/	233
CTCF	512	82386	184	8	53094	685	/	249	/	54
GTF2F1	1024	40399	573	22	37024	1776	/	633	/	148
RAD21	362	111671	158	7	51692	629	/	256	/	61
REST	724	57598	287	14	29816	1213	/	455	/	104

To the extent that some of the local maxima correspond to "noise" in the density estimate, we can say that larger kernel bandwidths and/or adaptively chosen bandwidths (as opposed to fixed bandwidths) generally produce less noisy density estimates. Specifically, among the three kernel functions considered, the square kernel tends to yield the most noisy signal, understandably due to its abrupt transitions (a rising edge and a falling edge for every kernel centered at a read). In comparison, the triangle kernel is smoother (or less noisy) due to its piecewise linearity. The Gaussian kernel, on the other hand, yields the smoothest (or the least noisy) density signal, due, of course, to its well-known smoothing and denoising properties [36].

### Adaptive-bandwidth KDE outperforms fixed-bandwidth KDE on held-out data

To more formally assess the accuracy of different density estimation strategies, we randomly divided each dataset into three parts: 50% for training (i.e., creation of the density estimate), 25% for tuning (setting parameters such as bandwidth *h *or number of neighbors *k*), and 25% for testing. We first focus on the results for Rad21, which are largely representative of the other datasets, before presenting a comparison across all ten datasets.

**Figure 3:**
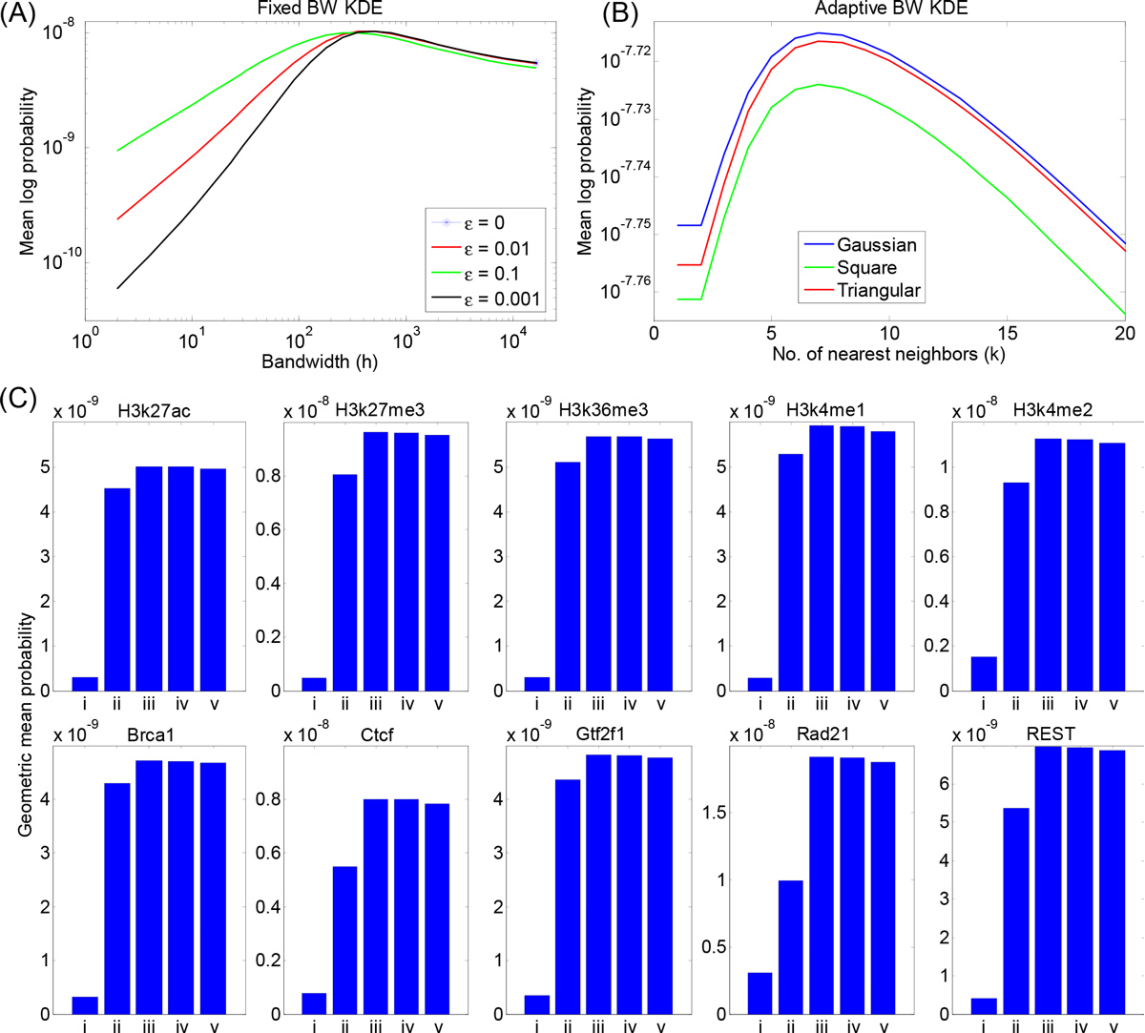
**Held-out tuning data analysis**. Assessment of (A) fixed and (B) adaptive bandwidth schemes on held-out tuning data from the Rad21 dataset across a range of the bandwidth parameter *h *and the *k_th_*-nearest neighbor parameter *k*. (C) Comparison of five different approaches on held-out test data from 10 ENCODE datasets: (i) fixed-bandwidth Gaussian with *h *= 16, (ii) fixed-bandwidth Gaussian with *h *optimized on the tuning set, (iii, iv, v) adaptive-bandwidth Gaussian, triangle, and square kernel functions with *k *optimized on the tuning set.

Figure 3A shows the results of several fixed-bandwidth density estimators on the Rad21 dataset: the standard fixed-bandwidth Gaussian kernel estimate (*ε *= 0), and that same estimate blended with a 10%, 1%, and 0.1% uniform density (*ε *= 0.1, 0.01, and 0.001, respectively). The vertical axis shows the mean log probability of the tuning data under the density estimate obtained using the training data. The horizontal axis shows the effect of varying the bandwidth. [The mean log probability of the tuning data is equivalent to the logarithm of the geometric mean of the tuning data point probabilities. We use the logarithm here for greater visibility of the plots. We employ the mean across tuning points so that different datasets with different total numbers of points can be compared directly.]

For the *ε *= 0 case, it is only at the largest tested value of the bandwidth parameter, *h *= 2^15 ^= 32768, that the tuning data has a nonzero probability. For smaller bandwidths, some tuning data points are left uncovered by any kernel in the training density. Such points get assigned a zero probability individually and, therefore, the entire tuning set is assigned a zero probability. For a typical, point-binding transcription factor, the peaks in the density may be a few hundred base pairs wide, and therefore smoothing with a kernel bandwidth in the tens of thousands is not ideal. In such situations, then, the vast regions of low signal levels demand a bandwidth inappropriate to the more interesting parts of the signal, and therefore choosing a single bandwidth becomes difficult.

For the *ε >*0 cases, the uniform density component solves the "zero problem" of tuning points being left uncovered (similar to our previous use of a uniform mixture component in analyzing multi-modal flow cytometry data [37]). The tuning data thus has nonzero probability for all choices of bandwidth, and by varying the bandwidth we can choose an appropriate one, as shown in Figure 3A. For this dataset, an appropriate bandwidth appears to be in the range of 500 to 1000 base pairs. We note that the best choice of bandwidth has some dependence on the *ε *parameter. Moreover, different choices of *ε *lead to mildly differing probabilities of the tuning data if bandwidth is optimized. For all choices of *ε*, however, we clearly see that too small a bandwidth leads to undersmoothing of the data, as seen by very poor probability of the tuning data. When bandwidth is too large, oversmoothing results, and the probability of the tuning data also suffers, although this loss is not as severe as that with undersmoothing. For other datasets, we often found that the tuning curves were even flatter for high bandwidths than the Rad21 tuning curve, rendering these datasets relatively resistant to oversmoothing. For the remainder of our analyses, we focus on the *ε *= 0.1 choice. Although the tuning data were slightly less probable with this choice than with smaller values of *ε*, this choice favored smaller bandwidths *h*, which is preferable for emphasizing regions of true signal density.

Figure 3B shows the results of adaptive-bandwidth density estimators with Gaussian, triangle, and square kernel functions for varying values of the nearest-neighbor parameter *k*. These results are again for the Rad21 dataset, and show the mean log probability of the tuning data under the density estimate obtained from the training data. We have used the KNN2 rule for assigning bandwidths. For this dataset, we find that an optimal value of *k *= 7 can be chosen based on the tuningset probability. Values smaller than this optimal value result in undersmoothing, and larger values result in oversmoothing. However, in absolute terms, the tuning-set probability actually changes very little as a function of *k*. The probability using the best value of *k *(7) is only about 10% higher than under the worst value of *k *tested. By way of comparison, better or worse values of the bandwidth parameter *h *for the fixed-bandwidth estimators in Figure 3A resulted in orders-of-magnitude differences in the tuning-set probability. We also observe that the probability of the tuning data obtained under the adaptive-bandwidth scheme is higher than that obtained under the fixed-bandwidth scheme. Intuitively, the adaptive-bandwith scheme allows, by design, coverage of sparse data regions without sacrificing accuracy in high-density regions. The Gaussian and triangle kernels performed very similarly, with the Gaussian being slightly better at all values of *k*. The square kernel fared slightly worse, although the difference is small compared to even the small loss that may result from a poor choice of *k*, let alone the difference observed for fixed-bandwidth density estimators.

Figure 3C compares the results of fixed and adaptive-bandwidth approaches on the full suite of the 10 datasets. We compare fixed-bandwidth Gaussian kernel estimation with *ε *= 0.1 and *h *= 16 (close to the *h *= *σ *= 15 choice that is the default in the QuEST software [23]), fixed-bandwidth Gaussian kernel estimation with *ε *= 0.1 and bandwidth *h *optimized on the tuning set, and adaptive-bandwidth estimation with Gaussian, triangle, and square kernel functions with KNN2 bandwidth selection and optimal *k *(chosen to maximize tuning-set probability). We report geometric mean probability of the test data points in the bar charts, while Table shows the optimized bandwidths *h *or nearest neighbor parameters *k*, depending on the method. The results are remarkably consistent across the 10 datasets. Adaptive-bandwidth estimation with Gaussian kernels is uniformly the best performer, followed closely by the triangle and the square kernels. Thus, by the measure of mean log probability of test data, the Gaussian kernel is consistently best at smoothing (or denoising) the data. Among the two fixed-bandwidth cases, the approach of optimizing bandwidth on a tuning set always leads to improved test-set performance, emphasizing the importance of using a tuning set to optimize algorithm parameters. For many datasets, the fixed (but optimized) bandwidth Gaussian estimator is only about 10% worse than the adaptive-bandwidth schemes, although for some datasets its performance drops to about two-thirds or even one-half of that of the adaptive-bandwidth schemes. The unoptimized fixed-bandwidth scheme is uniformly the worst, with a test-set probability on average about one-tenth of that of the other schemes.

### Kernel function choice influences time and space complexity

Although our analysis shows that the choice of the kernel function--Gaussian, square, or triangle--has little influence on tuning or test-set probabilities, the choice does impact the computational resources needed to compute the densities. The final columns of each half of Table show the CPU times, measured in seconds on a SunFire x2250 cluster computing node, for evaluating the full density estimate across chromosome 1 for the three different kernel functions. The Gaussian estimate is always the most expensive to compute, and there are two main reasons for this. First, it has the widest support of all the kernels (10 standard deviations in diameter), and it involves evaluation of the exponential function, which is a relatively time-consuming operation. The triangle estimate, with a smaller support and a simple function to evaluate, was typically about 2.5 times faster to compute. The square estimate, with the narrowest support and a constant height (though dependent on bandwidth), was roughly 10 times as fast to evaluate as the Gaussian estimate.

In slightly more formal terms, if we have *D *training data points, *S *base pairs of average kernel support, and a genome of size *G *base pairs, then we expect *O*(*DS *+ *G*) computations to evaluate a kernel density estimate. The *G *term is for initializing the density to zero everywhere, and the *DS *term is for evaluating each kernel over the base pairs to which it contributes probability mass. Empirically, the linear influences of *D *and *S *are well born out when we plot, for instance, CPU time versus dataset size or mean bandwidth size. Different kernel functions affect mainly the slope of the relationship of CPU time to *D *or *S*--i.e., they determine the constant inside the big O.

This analysis, however, assumes explicit representation of the density value at every base pair. The square kernel density estimate is piecewise constant, comprising *O*(*D*) pieces--each data point contributes one rise and one fall to the function $\hat{f}\left(x\right)$. Thus, the density can be represented in terms of the start, end, and height of each piece, and can be computed in *O*(*D*) time and requires only *O*(*D*) space (as opposed to *O*(*G*) space for a general density over the whole genome). Moreover, such a piecewise constant density is readily represented as a BED file, making it convenient for browser viewing. For the triangle kernel function, the density estimate is piecewise linear with *O*(*D*) pieces; this too can be handled in *O*(*D*) time and space, although we know of no browser file format allowing piecewise linear functions. Given that the triangle kernel has similar computational requirements to the square kernel, and yet an accuracy comparable to the Gaussian kernel, defining a browser file format that allows for piecewise linear functions could be advantageous. Thus, although accuracy points to the Gaussian kernel as the best choice for density estimation, square and triangle kernels have points in their favor regarding computation and browsing convenience.

## Conclusions and future work

We have investigated adaptive-bandwidth kernel density estimators for the reconstruction and visualization of genomic signals underlying ChIP-Seq data, with several results. First, we found that adaptive-bandwidth schemes generally outperform fixed-bandwidth schemes in terms of accuracy. In our opinion, adaptive-bandwidth schemes also hold visualization advantages, although we admit this is somewhat subjective. With optimized smoothing parameters, fixed-bandwidth estimators held a slight advantage in terms of computation time, although all estimates can be computed quickly enough that computation time does not seem to be a major concern. Among different kernel functions, we found that all yielded comparable accuracy with some having potential advantages in terms of compact representation and genome browser compatibility. It remains to be investigated whether the increased accuracy of adaptive-bandwidth estimates will translate into improved abilities of extracting biological information--for instance, in terms of transcription factor binding sites or peaks, assessment of regions of enrichment for histone marks or, more generally, comparison of different ChIP-Seq signals. Relatedly, our methods may be useful in more accurately decomposing genomic signals into constituent parts, or correcting for different sources of bias. One way to do this would be to create adaptive-bandwidth KDE smooths of different possible sources of bias (e.g., local GC content, mappability, etc.), and then use regression, deconvolution, or principal components style anlayses to isolate the true signal of interest. Alternatively, one might generalize the adaptive-bandwidth KDE approach to that of conditional density estimation to account for possible biases at the time of signal reconstruction. We leave these questions as topics for future study.

Software implementing adaptive-bandwidth density estimation for ChIP-Seq data is available at http://www.perkinslab.ca/Software.html.

## Competing interests

The authors declare that they have no competing interests.

## Authors' contributions

PR and TJP conceived the ideas, designed the experiments, and wrote and approved the paper.

## Declarations

Funding for the publication of this article was provided by the '*Author Fund in Support of Open Access Publishing *' established by the University of Ottawa.
